# The immune system and the impact of zinc during aging

**DOI:** 10.1186/1742-4933-6-9

**Published:** 2009-06-12

**Authors:** Hajo Haase, Lothar Rink

**Affiliations:** 1 Institute of Immunology, Medical Faculty, RWTH Aachen University Pauwelsstrasse 30, 52074 Aachen, Germany

## Abstract

The trace element zinc is essential for the immune system, and zinc deficiency affects multiple aspects of innate and adaptive immunity. There are remarkable parallels in the immunological changes during aging and zinc deficiency, including a reduction in the activity of the thymus and thymic hormones, a shift of the T helper cell balance toward T helper type 2 cells, decreased response to vaccination, and impaired functions of innate immune cells. Many studies confirm a decline of zinc levels with age. Most of these studies do not classify the majority of elderly as zinc deficient, but even marginal zinc deprivation can affect immune function. Consequently, oral zinc supplementation demonstrates the potential to improve immunity and efficiently downregulates chronic inflammatory responses in the elderly. These data indicate that a wide prevalence of marginal zinc deficiency in elderly people may contribute to immunosenescence.

## Review

### Introduction

The human body contains 2–3 g zinc, most of which is bound to proteins. Over 300 enzymes have been shown to contain zinc, either directly involved in catalysis, as a cofactor, or for structural stabilization [1]. Another large group of zinc containing proteins are transcription factors, many of which contain zinc fingers and similar structural motives. From *in silico *studies searching for known zinc-binding patterns, it has been estimated that approximately 10% of the human genome encode for proteins that could bind zinc [2].

Severe zinc deficiency is characterized by growth retardation, skin lesions and impaired wound healing, hypogonadism, anemia, diarrhea, anorexia, mental retardation, and impaired visual and immunological function [3,4]. Notably, also during milder forms of zinc deficiency an effect on immunity is observed.

On the cellular level, zinc is essential for proliferation and differentiation, but zinc homeostasis is also involved in signal transduction [5,6] and apoptosis [7]. Cells depend on a regular supply of zinc and make use of a complex homeostatic regulation by many proteins [8], but the plasma pool, which is required for the distribution of zinc, represents less than one percent of the total body content [1]. Despite its important function, the body has only limited zinc stores that are easily depleted and can not compensate longer periods of zinc deficiency. Additionally, during infections pro-inflammatory cytokines mediate changes in hepatic zinc homeostasis, leading to sequestration of zinc into liver cells and subsequently to hypozincemia [9]. Alterations in zinc uptake, retention, sequestration, or secretion can quickly lead to zinc deficiency and affect zinc-dependent functions in virtually all tissues, and in particular in the immune system.

### Role of zinc in the immune system

The trace element zinc is essential for growth and development of all organisms and the high rate of proliferation and differentiation of immune cells necessitates a constant supply with sufficient amounts of zinc. In the following section, we will discuss the different roles of zinc in the immune system.

In a review by Beisel, the effects of zinc deficiency on immunity in animal models are summarized [10]. The effects are hypoplasia of lymphoid tissues, and reductions in T-helper cell numbers, NK cell activity, antibody production, cell mediated immunity, and phagocytosis [10]. In humans, the most prominent example for the effects of zinc deficiency is *acrodermatitis enteropathica*, a rare autosomal recessive inheritable disease that causes thymic atrophy and a high susceptibility to bacterial, fungal, and viral infections [11]. It is a zinc-specific malabsorption syndrome based on a mutation within the gene for the intestinal zinc transport protein hZip4 [12,13]. All symptoms can be reversed by nutritional supplementation of excess zinc. Zinc deficiency does not affect just a single component of the immune system; the effects are complex, occur on many levels, and involve the expression of several hundred genes [14,15]. Short term effects include the regulation of the biological activity of thymulin by the plasma zinc status, while long term effects can lead to changes in immune cell subpopulations [16]. Even epigenetic effects were observed [17]. Gestational zinc deficiency in mice not only depressed the immune function of the offspring of these mice, but to a lesser extent compromised immune function was still found in the second and third filial generation, even though these mice had been fed with a zinc sufficient diet [17].

**Figure 1:**
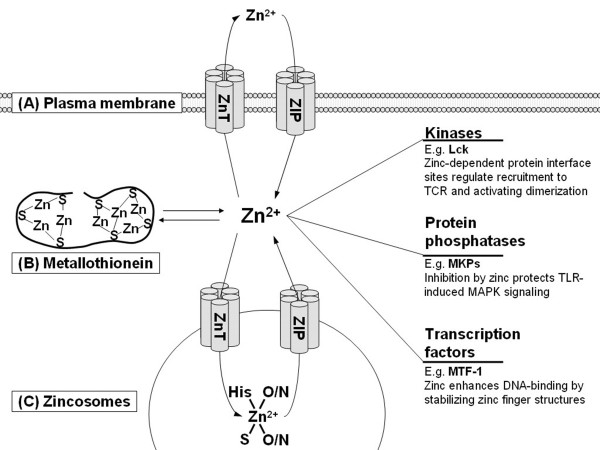
**Zinc as a signal molecule for immune cells**. Zinc homeostasis is tightly controlled by three mechanisms: (A) Transport through the plasma membrane by zinc transporters from the ZnT (SLC A30) or ZIP (SLC A39) families. (B) Buffering by metallothionein. (C) Reversible transport by ZnT and ZIP proteins into or out of zincosomes, and storage bound to ligands that form a zinc sink. Zinc signals, i.e., changes in the intracellular concentration of free zinc, control immune cell signal transduction by regulating the activity of major signaling molecules, including kinases, phosphatases, and transcription factors. One representative example for each group is given. (TCR, T cell receptor; MKP, MAPK phosphatase; MTF-1, metal-response element binding transcription factor-1).

One major mechanism by which zinc affects immunity is its role as a signaling ion (figure 1). The intracellular concentration of free zinc is regulated by three mechanisms. One is transport through the plasma membrane [5]. Another mechanism involves storage in and release from vesicles, so-called zincosomes, in which zinc is stored as a complex with multiple ligands [18]. Finally, zinc binds to metallothionein (MT). Through its 7 binding sites with different affinities, MT buffers zinc in the pico- to nanomolar range, and can additionally be controlled by release of zinc by oxidation of zinc-binding cysteine thiol residues [19].

Zinc signals, i.e. changes in the intracellular concentration of free zinc mediated by these three mechanisms, act on immune cell signal transduction [20]. The first example was protein kinase C (PKC), which has been identified as a molecular interaction partner for zinc in T cells [21]. Its N-terminal regulatory domain contains four Cys_3_His zinc binding motifs. Zinc treatment stimulates PKC kinase activity, its affinity to phorbol esters, and binding to the plasma membrane and cytoskeleton. Furthermore, zinc chelators inhibit the induction of these events by physiological activators of PKC [20].

The lymphocyte protein tyrosine kinase (Lck), a Src-family tyrosine kinase, is an example for a different mechanism by which zinc acts on signal transduction. Zinc ions promote activation of Lck and its recruitment to the T cell receptor complex by linking two protein interface sites. The N-terminal region of Lck is recruited to the intracellular domains of the membrane proteins CD4 or CD8 by a 'zinc clasp' structure [22-24]. At the second zinc-dependent interface site two zinc ions at the dimer interface of the SH3 domains stabilize homodimerization of Lck, which is thought to promote autophosphorylation required for its activation [25].

Zinc signals were also observed when monocytes were treated with lipopolysaccharide. These zinc signals regulate inflammatory signaling [26]. Here, cyclic nucleotide phosphodiesterases and MAPK phosphatases were identified as molecular targets of zinc [26-28]. Signaling via the transcription factor NF-κB is also dependent on zinc signals; however, in this case it is no direct interaction with zinc, but rather a regulation of upstream signaling pathways leading to the activation of NF-κB [26].

Recent papers demonstrate an influence of zinc transporters on signal transduction. Zrt/Irt-like protein (ZIP)7 releases Zn from the ER, controlling tyrosine phosphorylation [29], and lysosomal ZIP8 is required for zinc-mediated calcineurin inhibition and interferon (IFN)-γ expression in T cells [30]. Conversely, there also exist feedback mechanisms, which act on zinc homeostasis. The promoters of MT and of several zinc transporters are under the control of the metal-response element binding transcription factor (MTF)-1. In contrast to other transcription factors with zinc fingers that bind zinc constitutively, its DNA-binding is regulated by the stabilization of zinc finger motifs by free cellular zinc [5,31,32].

Zinc deficiency in the elderly may impair zinc-dependent signaling, and thereby immune function. In one recently published study, peripheral blood mononuclear cells (PBMC) from zinc-deficient elderly showed impaired NF-κB activation and interleukin (IL)-2 production in response to stimulation with PHA, which was corrected by *in vivo *supplementation of zinc (45 mg/day as gluconate) for 6 months or *ex vivo *supplementation of zinc to PBMC [33], indicating a link between zinc deficiency and the effect of zinc on NF-κB signaling.

#### Zinc and innate immunity

Zinc supplementation *in vitro *can trigger events required for the recruitment of leukocytes to the site of infection. For example, high zinc concentrations induce chemotaxis of polymorphonuclear cells [34], and zinc promotes the adhesion of myelomonocytic cells [35]. On the other hand, zinc deficiency *in vivo *causes impaired phagocytosis, parasite killing, and oxidative burst of monocytes and neutrophil granulocytes, and a decrease in NK cell activity [36-38]. Zinc is also required for recognition of HLA-C molecules by the killer cell inhibitory receptors on NK cells, but, notably, zinc is only necessary for inhibitory, but not stimulatory effects [39]. Via this mechanism, zinc deficiency may promote nonspecific killing by NK cells. However, this effect is counteracted by a reduction of NK cell lytic activity in zinc deficient patients [40].

#### Zinc and adaptive immunity

The adaptive immune response is based on two groups of lymphocytes: B cells, which differentiate into immunoglobulin secreting plasma cells and hereby induce humoral immunity, and T cells, which mediate cytotoxic effects and helper cell functions of cell mediated immunity. Both responses depend on the clonal expansion of cells after recognition of their specific antigen. While B cells depend on zinc for proliferation, they do so to a lesser extent than T cells [41,42]. In addition, a heightened level of apoptosis in pre B and T cells was found in zinc deficient mice. Mature cells are more resistant to apoptosis induced by zinc deficiency, possibly because of the higher level of the anti-apoptotic protein BCL-2 in these cells [16]. Not only does zinc deficiency affect B cell lymphopoiesis, it has also been shown to lead to a reduction in antibody-mediated immune defense [16].

The most prominent effect of zinc deficiency is a decline in T cell function, which results from multiple causes. Thymulin, a hormone secreted by thymic epithelial cells, requires zinc as a cofactor and exists in the plasma in two forms, a zinc-bound active one, and a zinc-free, inactive form. It is essential for differentiation and function of T cells, which could explain some of the effects of zinc deficiency on T cell function. In mice, zinc deprivation reduces the level of biologically active thymulin in the circulation [43]. This effect has been observed in the absence of thymic atrophy, and thymulin activity was restored after *in vitro *supplementation of the serum with zinc, indicating that thymulin activity is directly dependent on serum zinc [44]. In mildly zinc deficient humans, thymulin activity was also decreased, and a comparable effect of zinc supplementation *in vitro *and *in vivo *was described [45].

Furthermore, the TH1/TH2 balance is affected by zinc. During zinc deficiency, the production of TH1 cytokines, in particular IFN-γ, IL-2, and tumor necrosis factor (TNF)-α is reduced, whereas the levels of the TH2 cytokines IL-4, IL-6, and IL-10 were not affected in cell culture models [46] and *in vivo *[47,48]. In addition to the immunomodulatory effects of zinc deprivation, zinc supplementation can modulate T cell dependent immune reactions. Zinc supplementation to PBMC leads to T cell activation, an indirect effect that is mediated by cytokine production by other immune cells, but higher concentrations of zinc can also directly suppress T cell function. Here, zinc reduces IL-1 dependent T-cell stimulation by inhibiting the interleukin-1 receptor associated kinase-1 [49]. *In vitro*, zinc inhibits the mixed lymphocyte culture (MLC) [50], and a clear reduction in the MLC was also shown in PBMC from human subjects that had been supplemented with 80 mg zinc per day for one week. Notably, the response to a recall antigen, tetanus toxoid, was unaffected in these cells and zinc specifically inhibited the allogenic reaction [51].

#### Zinc and cytokine levels

Zinc has been characterized as a positive and negative regulator of pro-inflammatory cytokines, in particular IL-1 and TNF-α. Some reports describe that zinc supplementation to human peripheral blood mononuclear cells leads to an increased mRNA production and release of the monokines IL-6, IL-1β, and TNF-α, and a combination of nonstimulatory concentrations of LPS and zinc results in the production of large amounts of monokines [52]. On the other hand, several reports indicate that zinc treatment suppresses the formation of pro-inflammatory cytokines [46,53]. This difference can be explained by the observation that the effect of zinc is concentration dependent, and that zinc can be stimulatory or inhibitory in the same experimental system. Whereas an increase of intracellular free zinc, which can be imitated by moderate zinc supplementation to cell cultures, is a zinc signal involved in cytokine production of monocytes in response to LPS [26], higher concentrations can have an antagonistic effect by inhibition of cyclic nucleotide phosphodiesterases and a subsequent activation of protein kinase A [27,28]. In T cells, cytokine secretion is only indirectly affected by zinc. Zinc-induced release of IFN-γ and the soluble IL-2 receptor depends on the presence of monocytes, and is based on direct cell to cell contact and zinc-mediated production of the monokines IL-1 and IL-6 [52].

### Immunological changes during aging

Aging of the immune system, also referred to as immunosenescence, describes the age-related changes in immune function that lead to increased susceptibility of older people to infectious diseases, autoimmunity, and cancer. The capacity of the immune system to mount an adequate response decreases with age, starting around 60, but several factors such as lifestyle and underlying diseases can significantly affect the onset in each individual [54]. Interestingly, a comparison between alterations of the immune system during zinc deprivation and aging shows many similarities, indicating a possible relation between immunosenescence and zinc deficiency [55]. In both cases it comes to anergy, thymic atrophy, and reduced NK cell activity, cell mediated cytotoxicity, helper T cell activity and thymulin levels [56].

As it could be expected from the decline in immune function, aged patients suffer from an augmented incidence and mortality of infectious diseases such as pneumonia [57] and tuberculosis [58], and re-infections with herpes zoster increase [59]. The frequency of autoimmune diseases is augmented with age, too, accompanied by an increase in autoantibodies, which is, interestingly, not observed in centenarians [60,61]. On the other hand, specific IgE production decreases, reducing the risk for allergies [62,63].

Cancer is a disease that occurs over proportion in elderly as well. People ≥65 years have an eleven fold higher incidence of cancer and a fifteen fold higher mortality than younger subjects [64]. Although the immune system functions as a network in which nearly all elements interact with each other, some components can be identified that are especially affected by aging and whose functional impairment causes increased susceptibility for diseases like the examples mentioned above [65,66].

Neutrophil granulocytes form the first line of defense against pathogens, mainly by phagocytosis, but also cytokine secretion and recruitment of other immune cells. The higher incidence of microbial infections in the elderly, although often attributed primarily to a decline in T cell function, may also in part be the result of an impairment of neutrophils. The total number of neutrophils is not different in the aged compared to younger controls. However, phagocytosis, oxidative burst, and intracellular killing are affected and neutrophils from the elderly show a reduction in chemotaxis and a reduced resistance toward apoptosis, based on a diminished antiapoptotic effect of stimuli such as LPS, G-CSF, and GM-CSF [67].

While the activity of all other immune cells decreases with age, some functions of macrophages, and their precursors monocytes, are even augmented in elderly. No change in the number of monocytes in the blood is observed and, in contrast to neutrophils, chemotaxis, phagocytosis, and oxidative burst remain unchanged [68]. However, their accessory function for T cells is impaired, although the expression of several cytokines, adhesion molecules, and HLA-DR is not altered [69]. The plasma concentrations of IL-6, IL-8, MCP-1, MIP-1α, and TNF-α are positively correlated with age [70]. Furthermore, production of pro-inflammatory cytokines such as IL-1, IL-6, IL-8, and TNF-α after stimulation with LPS is significantly increased [71,72]. In contrast, IFN-α, which is mainly produced by monocytes, is reduced [73]. The other major group of antigen presenting cells, dendritic cells, seem to be unaffected by age with respect to surface marker expression and transendothelial migration [74]. The total number of NK cells and their percentage among circulating cells is increased in old people, but this effect is compensated by a reduced cytotoxic activity on a per-cell basis and reduced proliferation in response to IL-2 [75-77], together with reduced calcium signaling and CD69 expression, while TNF-α secretion remains unaffected [78]. Because the main functions of NK cells are the elimination of cancer or virus infected cells, the higher incidence of viral infections and cancer in the elderly may well be related to impairment of NK cell function.

**Figure 2:**
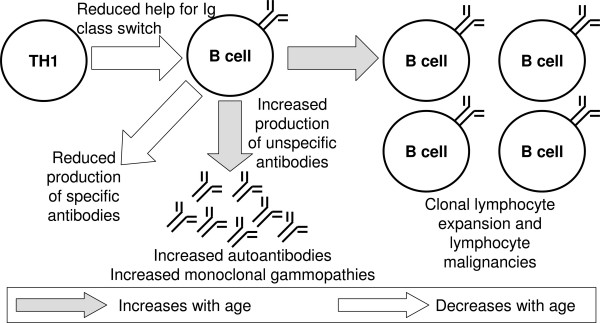
**Disturbed B-cell function in ageing**. In general, the numbers of B cells and specific antibodies (e.g., in response to vaccination) decrease with age, while total and unspecific immunoglobulin and autoantibodies increase. Some B cell clones expand, resulting in higher probability for lymphocyte malignancies.

The most severe changes during aging are found in the adaptive immune system. Aging leads to a shift in B cell populations and antibody production. B cell numbers decline with age and one would expect that this is accompanied by a decrease in immunoglobulins, but the opposite has been observed, showing an increase of IgA and several IgG subclasses [79]. The response to vaccination with several antigens is diminished, which may result from an impaired interaction with T helper cells (see below), but also a loss of antibody affinity was found. At the same time an increase in organ-specific and non-organ-specific autoantibodies was observed, but, whereas the latter increase with age, subjects over 90 years show lower levels of organ-specific autoantibodies than younger elderly [80]. Another change that occurs with age is increased clonal expansion of B cells, which may be connected to the increased incidence of lymphocyte malignancies with age [80]. The effects of aging on B cells and humoral immunity are summarized in figure 2.

Similar to the effects of zinc deficiency, the main changes of aging also affect the T cell system. T cells from elderly subjects show decreased proliferation in response to T cell receptor (TCR) stimulation or mitogens [81], an altered CD4/CD8 ratio, and higher expression of CD95 and the pro-apoptotic BAX combined with a decrease in BCL-2 and p53, which leads to increased apoptosis [82]. A prominent feature of immunosenescence is thymic involution. This leads to a decrease in the generation of new T cells, finally resulting in a lower number of naïve (CD45RA+) and a higher number of memory (CD45R0+) T cells [83]. Zinc deficiency can also cause thymic involution, regardless of age. A reduction of zinc availability induces higher levels of thymocyte apoptosis, either by elevating glucocorticoid production or because zinc has a negative regulatory function in immune cell apoptosis [16,20,84]. Notably, supplementation of the drinking water of old mice with zinc sulfate has been reported to induce an increase in thymic mass [85], and parameters such as thymic weight, the number of viable thymocytes, and serum thymulin activity were restored by oral zinc supplementation [86]. Hence, lower zinc levels in the elderly could contribute to thymic involution by augmenting apoptosis during T cell maturation and selection in the thymus.

As in B cells, monoclonal expansion has been found for T cells from elderly subjects. The expanded subsets can make up a large fraction of T cells, but no signs of malignant transformation have been reported [87]. The expanded subsets were primarily CD8 positive whereas CD4 populations remained unchanged. However, T helper cells are also affected by aging, showing a decreased TH2/TH1 ratio in the elderly, measured by CCR4/CCR5 surface expression [88]. In addition, alterations in the balance of TH1/TH2 cytokines occur that are similar to the effects observed during zinc deprivation [88]. The TH1 cytokines IFN-γ, IL-2, and sIL-2R are reduced. In contrast, TH2 cytokines IL-4 and IL-10 are increased, resulting in a shift toward TH2 cytokines [89,90].

**Figure 3:**
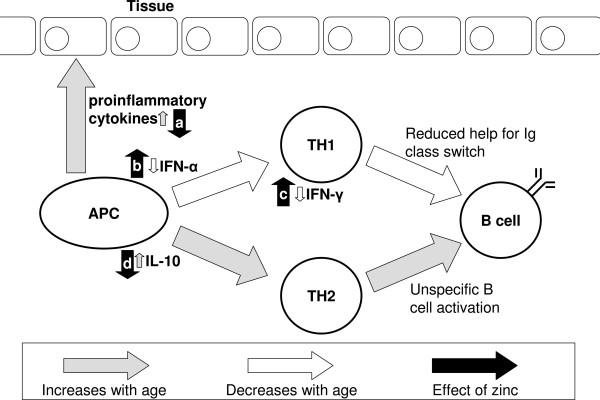
**Influence of zinc on age-related changes of immune function**. Aging leads to an increase in pro-inflammatory cytokines and modulates the TH1–TH2 balance toward a TH2 response by reducing the TH-1 cytokines IFN-α and -γ and increasing IL-10. This reduces T cell help for immunoglobulin class switch and causes unspecific activation of B cells. Zinc counteracts the effects on [a] pro-inflammatory cytokines [27], [b] IFN-α [73], [c] IFN-γ [92], and [d] IL-10 [93].

Decreased humoral immunity may not only result from changes in B cells, but in part be caused by a disturbance of T cell help and alterations of cytokine levels, because many cytokines that control B cell functions are affected by aging [90,91]. As summarized in figure 3, a disturbed humoral response may be the result of a combination of an impaired interaction between antigen presenting cells (APC) and T helper cells and a shift in the TH1/TH2 balance, which both add to the immunological alterations that occur directly in B cells. It is noteworthy that zinc can antagonize all these effects: Zinc supplementation can suppress the release of pro-inflammatory cytokines from LPS-stimulated monocytes [27], and addition of zinc to PBMC promotes IFN-γ release [92]. *In vitro *zinc supplementation can also decrease IL-10 release [93] and restore IFN-α production from leukocytes of elderly subjects [73]. However, the effect of zinc is not limited to cytokine expression, because the antiviral activity of IFN-α, but not IFN-β and -γ, is potentiated by addition of zinc *in vitro *[94].

### Zinc status of the elderly

Many micronutrients affect immunity and suboptimal nutritional supply can cause an impaired immune response [95]. This is especially true for zinc, given its essential role in many immunological processes, as described above. In many elderly, the required supply of zinc is not met [96]. A multitude of influencing factors has been suggested, which include physiological, social, psychological, and economic factors. For example, reduced mobility leads to a decrease in energy requirements. The resulting consumption of smaller quantities of food also means consuming lower amounts of trace elements, including zinc. In addition, decreased intestinal absorption, which in part depends on the composition of the food, and medication like diuretics, could cause a negative zinc balance, even if there is sufficient uptake. All these factors together can result in insufficient nutritional supply with trace metals in the elderly [4]. Finally, some diseases that occur with increased frequency in older people, such as diabetes, are also accompanied by zinc deficiency [4,97,98].

The recommended daily allowance (RDA) for zinc in individuals 19 years and older (special recommendations for elderly do not exist) in the United States is 11 mg/day for men and 8 mg/day for women [98]. An uptake below the RDA can only be seen as an indicator of potential zinc deficiency, because many other factors also play a role and the possibility exists that the metabolism may adapt to decreased zinc intake. Hence, it is necessary to analyze the zinc status of the individual. The parameter of choice is often serum or plasma zinc. However, this is not an ideal parameter for determining the zinc status. When zinc deficiency was experimentally induced in young subjects, they showed significant effects on the production of IFN-γ, IL-2, and TNF-α with an imbalance in the TH1/TH2 system, but plasma zinc was not significantly affected [47]. Whereas reduced plasma or serum zinc levels can indicate zinc deficiency, such deficiency can also occur at levels that are within the reference values [98]. Possibly, other parameters, such as labile intracellular zinc in leukocytes, will be a more accurate measure for the zinc status in the future [99].

Many studies about zinc nutrition and status in the elderly exist (table 1). In most of these studies, zinc deficiency is defined as a serum or plasma concentration below 10.7 μM, which corresponds to 70 μg/dL. In most cases a clear tendency toward suboptimal zinc intake and decreasing zinc plasma and serum levels with age were found, but values were still within the reference range of 70 – 110 μg/dL. An early study in 1971 investigated the correlation between age and plasma zinc in 204 male subjects between the ages of 20 to 84, and 54 female subjects between 20 and 58, finding a significant linear decrease of plasma zinc with age in both groups [100]. In contrast, red blood cell zinc content was even slightly increased, although these effects were not significant [100]. A significant reduction in serum zinc was also found for the 'oldest old' (≥ 90 years), compared to healthy elderly between 65 and 89 years and adults between 20 to 64 years [101]. Three other studies did also not find a high prevalence of zinc deficiency in the elderly, but while not being deficient, in one study mean plasma levels were low (<85 μg/dL) [102], in another serum zinc levels were significantly below a young control group [89], and erythrocyte zinc concentration was lower in 70–85 year olds, compared to a group between 55 and 70 years [103].

**Table 1:** Zinc status of the elderly.

**Subjects**	**Zinc status**	**Reference**
204 males, 20 – 84 y. 54 females, 20 – 58 y.	significant decrease of plasma zinc, but not erythrocyte zinc, with age	[100]

146 elderly, 65–95 y.	mean plasma levels below 85 μg/dL (= 13 μM)	[102]

121 elderly, 60–97 y.	Average zinc intake 7.3 mg/day, 6% had serum zinc under 70 μg/dL (= 10.7 μM)	[132]

24 healthy, 69–85 y. 50 controls, 21–64 y.	reduced plasma zinc compared to young controls	[106]

20 chronically ill elderly, 70–85 y.	compared to Bunker et al. 1984 no effect on plasma and whole blood zinc, but reduction of leukocyte zinc	[107]

100 elderly, 60–89 y.	14.7% zinc deficient (<10.7 μM, plasma), >90% had intake below RDA (15 mg/ml in 1987)	[123]

23 elderly, 65–85 y. 13 controls, 23–45 y.	IL-2 production was lower in elderly with reduced leukocyte and neutrophil zinc	[126]

232 hospitalized, 60–104 y. 25 free living, 69–94 y.	serum and leukocyte zinc lower in hospitalized subjects	[122]

53 healthy elderly, 64–95 y.	serum zinc decreases with age, mean serum zinc within normal range, 65% had intake less than 2/3 RDA	[105]

19 healthy, 51.3 m.a. 25 healthy, 77.7 m.a. 30 hospitalized, 80.8 m.a. 34 w/ulcers, 81.3 m.a.	plasma zinc negatively correlated with age, plasma and leukocyte zinc lower in hospitalized elderly compared to both healthy control groups	[121]

30 patients, 72–98 y. 12 healthy, 75–86 y. 23 controls, 18–55 y.	plasma zinc significantly decreased in both groups of elderly, zinc is lowered in polymorphonuclear but not mononuclear cells of elderly patients	[116]

118 subjects, 50–80 y.	decrease in lymphocyte and granulocyte zinc, ~30% defined as zinc deficient	[115]

21 elderly, 70–90 y. 20 young, 20–35 y.	significantly lower serum zinc in the elderly	[89]

81 hospitalized, 65–102 y.	61% of subjects zinc deficient (<10.7 μM)	[119]

345 elderly, > 70 y.	19% had hypozincemia (<12.2 μM), values of nursing home residents significantly lower than free living	[117]

29,103 subjects, NHANES III	42.5% of ≥71 y. had adequate zinc intake	[108]

62 healthy, 90–106 y.	zinc deficiency in 52% male and 41% female subjects, based on a reference range established in 20–64 y. controls	[112]

44 oldest old, 90–107 y. 44 elderly, 65–89 y. 44 young, 20–64 y.	serum zinc significantly reduced in oldest old compared to elderly and young	[101]

50 hospitalized, 83.5 m.a.	28% deficient (<10.7 μM serum zinc)	[118]

13,463 subjects, NHANES II	Correlation between serum zinc and age, decline starts at age 25	[104]

10 oldest old, 93–102 y. 15 old, 65–80 y. 15 young, 20–40 y. 10 infected, 63–75 y.	Significantly lower zinc in both groups of older subjects compared to younger ones, no decrease from old to oldest old Lowest levels found in infected patients	[113]

101 elderly, 56–83 y.	35% zinc deficient (<90 μg/dL plasma zinc)	[33]

668 hospitalized, 80.4 m.a. 105 healthy, 80.9 m.a.	20.2% zinc deficient (<70 μg/dL (or 10.7 μM) serum zinc) in the hospitalized, none in the healthy controls	[120]

188 aged, 55–70 y. 199 older 70–85 y.	Erythrocyte zinc lower and urinary zinc higher in the older participants. Less than 5% had insufficient zinc uptake (< 2/3 RDA)	[103]

93 healthy elderly, 55–70 y.	Average of 13.0 μM serum zinc	[134,139]

67 elderly, 71.7 m.a.	Mean serum zinc 61.8 μg/dL (= 9.4 μM), 76.3% zinc deficient (<70 μg/dL or 10.7 μM)	[114]

NHANES: National Health and Nutrition Examination Surveys, RDA: Recommended Daily Allowance, y.: years, m.a.: mean age

These findings have been confirmed by data from the second National Health and Nutrition Examination Survey (NHANES). In its course, over 13,400 serum samples were analyzed for their zinc content. Serum zinc levels increased into the third decade, and declined from there [104]. In combination with an age-dependent decrease of serum zinc, insufficient nutrition and low zinc intake were described, but again mean serum zinc was still in the normal range [105]. In another study, a significant difference between plasma zinc of healthy elderly and a young control group has been found and the average daily intake of healthy elderly was only 60% of the RDA [106], and even significantly lower in housebound chronically ill (39% RDA) [107]. This study describes no difference between plasma and whole blood zinc contents between healthy and chronically ill elderly people, but a significant reduction of leukocyte zinc [106,107]. The third NHANES has demonstrated in a large study population that inadequate zinc intake is frequent in American elderly [108], and similar observations are reported from other regions of the world, as well. The incidence of zinc deficiency also increases with age in the Japanese [109]. Furthermore, the European Nutrition and Health Report summarizes data regarding the nutritional zinc uptake in elderly from Austria, Denmark, Germany, Hungary, and the UK. Zinc supply decreases with age, although it can be generally regarded as sufficient. Furthermore, there is considerable variation between countries, and zinc uptake is particularly low in UK elderly [110].

Centenarians are a remarkable subgroup of the elderly, who have achieved 'successful aging', without suffering from age-related diseases [111]. Due to its beneficial effect on immunity and healthy aging, measuring the zinc status of these individuals seems indicated to investigate the potential contribution of a difference in zinc homeostasis to the greater health of centenarians. However, it was shown that healthy nonagenarians and centenarians have a high prevalence of zinc deficiency [112]. It still remains to be seen if the decrease of zinc levels reaches a constant level at a certain age, or if the decline continues after the eight decade of life. In one study, measurements showed a reduction in healthy 65–80 year old compared to the zinc status of young adults, but no further reduction in nonagenarians/centenarians [113]. In contrast, a comparison of serum zinc between subjects younger than 65 years, compared to ones aged 65–89 years, and to subjects ≥ 90 years showed a significant decrease between the oldest old an the other two groups indicating a continuing reduction [101].

These data indicate that improved immune efficiency that promotes successful aging in centenarians is not based on a difference in their zinc status, but act via an unrelated mechanism. This is in accordance with the observation that parameters that are associated with reduced zinc levels, e.g., increased production of pro-inflammatory cytokines, are still observed in centenarians [111].

Whereas it is a general finding that plasma and serum zinc decrease with age, few studies find a high frequency of zinc deficiency in the elderly. In one study, subjects 90 years and older were zinc deficient compared to reference data that the same laboratory had measured in younger individuals [112]. In 67 south African elderly with a mean age of 71.7 years, mean serum concentration was 61.8 μg/dL, with 76.3% of the study population being zinc deficient (<70 μg/dL) [114]. Another group found that only 42.9 percent of the elderly subjects that were investigated had a sufficient intake of zinc (>67% RDA) [115]. However, it has to be noted that in this and several other older studies higher RDAs of 15 mg (male) and 12 mg (female) were used. Even with the current, lower RDAs zinc deficiency would be frequent in the studied population, and 30% were also classified as zinc deficient based on their granulocyte and lymphocyte zinc content. Again, plasma zinc did not indicate zinc deficiency in these subjects, underscoring the difficulties with the use of this parameter [115].

The considerable variability in the classification of elderly people as zinc deficient, either according to their intake or measured zinc status, is caused by more than the use of different RDA, different parameters to measure the zinc status, or the use of different reference values to define zinc deficiency. They can also result from a limited comparability of the populations that are investigated. In addition to regional differences, which affect factors such as food composition, health status and living conditions have great influence. Many studies were performed with healthy elderly. If zinc is as important for immune function as indicated above, this group is the most likely to have normal zinc values. Hence, a difference is likely to exist between apparently healthy, free living elderly and institutionalized subjects, and this has already been described in studies that directly compare these groups [116,117]. Accordingly, a high prevalence of zinc deficiency was found in 50 hospitalized elderly patients, 28% of which were zinc deficient (<10.7 μM plasma zinc) [118], another group of 81 hospitalized subjects (65–102 years) whose mean serum zinc was below 10.7 μM, and 61% of which were zinc deficient [119], or in a study where 20.2% of hospitalized elderly (≥70 y.) had serum zinc below 70 μg/dL, while a healthy control group included no zinc deficient subjects [120].

Some authors speculate that insufficient intake or low zinc content in hospital diets may be responsible for the reduced zinc levels found in sick, and especially in hospitalized patients [121]. A negative overall zinc balance in housebound chronically ill patients was documented by a detailed metabolic balance study, in which an average intake of only 39% of the RDA was found [107]. However, in another study hospitalized elderly had reduced serum and leukocyte zinc levels compared to a free living control group of similar mean age, although their mean dietary intake of zinc did not vary significantly [122].

Independent from the classification of elderly as zinc deficient, correlations between zinc status and immunological parameters have been observed, indicating that even marginal zinc deficiency can affect immunity, while the zinc status is still within the reference values. A study by Bogden and coworkers demonstrates a positive correlation between plasma zinc concentration and delayed cutaneous response to skin antigens [123]. Hereby, even small differences of only 1.5 μM seemed to affect skin test anergy. In elderly hemodialysis patients, a correlation between Diphtheria vaccination and zinc status was described. Compared to age-matched controls, the group of patients who did not respond to vaccination had reduced serum zinc levels (p < 0.004), whereas the levels of responders were not significantly decreased [124].

Proliferation and cytokine secretion in response to stimulation with PHA were analyzed in lymphocytes isolated from healthy elderly (70–85 y.) subjects with mean zinc intake and serum and erythrocyte levels within the normal range. There was a positive trend for a correlation between proliferation and serum zinc in male subjects. Furthermore, the production of IL-10 in response to PHA showed a negative correlation with erythrocyte zinc in males, while baseline and PHA-stimulated production of this cytokine were negatively correlated with serum zinc in females [125].

Reduced IL-2 production upon stimulation with PHA was observed in elderly subjects who had reduced levels of cellular zinc in lymphocytes and neutrophils, whereas IL-2 production was not affected in zinc sufficient elderly and younger controls [126]. In another study, subjects 90 years and older were not only zinc deficient, but a positive correlation between serum zinc and percentage of NK cells among leukocytes was established [112]. In a different group of hospitalized patients, serum zinc was negatively correlated to IgG2 levels. Additionally, zinc deficient patients had significantly higher frequencies of congestive cardiopathy, respiratory infections, gastrointestinal diseases, and depression [118].

A decline of zinc status with age has been established, and a correlation between zinc status and immune function in the elderly seems to exist. The question remains if zinc deficiency is caused by infections that occur more frequently in elderly people and lead to a subsequent loss of zinc, or if aging poses a risk of becoming zinc deficient, leading to immunosenescence and increased susceptibility to infectious diseases. In the latter case, zinc supplementation could be a useful approach to improve the immune status of elderly people.

### Effect of zinc supplementation on elderly

Several studies have investigated the impact of zinc supplementation on the immune defense [127], and some of them focused on the investigation of the effect of zinc supplementation on different immune parameters particularly in elderly subjects. Their mean findings are summarized in table 2. The results are difficult to compare not only due to differences in the studied populations and their zinc status, but also due to study design, the immunological parameters that have been investigated, and dosage, duration, and bioavailability of zinc supplementation.

**Table 2:** Zinc supplementation studies in elderly.

**Subjects**	**Number**	**Intervention^1^**	**Effect**	**Reference**
institutionalized > 70 years	15 (C) 15 (Z)	100 mg zinc as sulfate one month	increased T cell numbers, DTH, and response to tetanus vaccine compared to control group	[130]

anergic to DTH, 64–76 years	5 (Z)	55 mg zinc as sulfate four weeks	improved DTH	[132]

free-living, 60–89 years	36 (P) 36 (Z,15) 31(Z,100)	15 or 100 mg Zn as acetate 3 months	no effect on DTH or *in vitro *lymphocyte proliferation	[137]

zinc-deficient males, 65–78 years	8 (Z)	60 mg zinc as acetate 4.5 months	increase in DTH after supplementation	[131]

free-living, 60–89 years	24 (P) 20 (Z,15) 19(Z,100)	15 or 100 mg Zn as acetate 12 months	negative effect on DTH, NK cell activation only after 3 months	[138]

institutionalized, 73–106 years	44 (P)/(Z) crossover	20 mg zinc as gluconate 8 weeks	increased thymulin activity	[136]

zinc deficient, 50–80 years	13 (Z)	30 mg zinc as gluconate 6 months	increase in plasma thymulin activity, IL-1, and DTH after supplementation	[115]

institutionalized, 64–100 years	190 (C) 160 (Z)	90 mg zinc as sulfate 60 days	no effect of zinc on response to influenza vaccination	[149]

institutionalized, ≥ 65 years	30 (P) 28 (Z)	25 mg zinc as sulfate 3 months	increase in CD4+DR+ T cells and cytotoxic T cells compared to placebo	[133]

free-living, 65–82 years	19 (Z)	10 mg zinc as aspartate 7 weeks	reduced levels of activated T helper cells and basal IL-6 release from PBMC, improved T cell response	[140,141]

institutionalized	25(P) 24(Z) 6(P) 6(Z)	45 mg as gluconate 12 months 45 mg as gluconate 6 months	reduced incidence of infections increased IL-2 mRNA in response to *ex vivo *stimulation with PHA	[33,128]

healthy, 55–70 y.	31 (P) 28/34 (Z)	15/30 mg zinc as gluconate 6 months	no effect on markers of inflammation or immunity	[134]

^1^The values are given as elemental zinc

DTH: delayed type hypersensitivity reaction, (C) control group without supplementation, (P) placebo, (Z) zinc supplementation

Several studies find a beneficial effect of zinc on human health. Zinc supplementation (45 mg elemental zinc as gluconate vs. placebo) to a group of elderly significantly reduced the incidence of infections during a one year course [128]. In another group of elderly, supplemented with a mixture of vitamins and minerals including zinc (7 mg per day, given as sulphate) for one year, the incidence of pneumonia was significantly higher in individuals with low (<70 μg/dL, corresponding to 30% of the study group) serum zinc, compared to ones that were not zinc deficient [129].

Multiple reports describe an effect of zinc on T cells of elderly subjects. In one of the first studies investigating the effect of zinc supplementation on the immune system, healthy subjects over 70 years of age received 220 mg zinc sulfate (corresponding to 50 mg of elemental zinc) twice daily for one month, and were compared to a control group that was not supplemented with zinc [130]. Zinc status was not assessed. Whereas the total number of circulating lymphocytes was not affected, the proportion of T cells was significantly increased, but this did not lead to a change of the response to *in vitro *stimulation with T-cell mitogens. An increased delayed type hypersensitivity (DTH) reaction and response to vaccination with tetanus toxiod was observed [130]. Three further studies confirmed the effect of zinc supplementation on DTH with lower zinc doses, but all were performed with a low number of participants and without a control group [115,131,132]. Wagner et al. investigated 5 subjects that were anergic to four different skin test antigens (*Candida*, *Trochophyton*, mumps, tuberculin), and all five tested positive to at least one antigen after 4 weeks of supplementation with 55 mg zinc (as sulfate) per day [132]. Cossack found in eight zinc deficient elderly males, who were classified as anergic to skin antigen tests, an improvement of DTH after supplementation with 60 mg per day. This was accompanied by an increase of plasma and cellular zinc [131]. Prasad and coworkers investigated 13 zinc deficient subjects whose plasma zinc levels and granulocyte and lymphocyte content increased significantly after supplementation with 30 mg zinc per day. They also found an increase in the number of positive skin test reactions after six months [115]. However, as no control groups have been investigated in either study, it can not be excluded that repeated testing may have contributed to the improvement in DTH reactions.

A beneficial effect of zinc on T cell function has also been observed when other parameters were investigated. A significant increase in the numbers of cytotoxic T cells and activated (HLA-DR positive) T helper cells was found in residents of a retirement home who had been supplemented with zinc (25 mg per day) [133]. This raise in HLA-DR positive cells seems to result from increased total T cell numbers, while the percentage of activated cells within the T cell population remains constant [133,134]. Fabris et al. have found decreased plasma zinc with age and an age-dependent decrease of plasma thymulin activity. Because thymulin activity was restored by *in vitro *addition of zinc, the effect was not caused by thymic involution, rather was thymulin inactive due to decreased plasma zinc [135]. This observation has been confirmed in a later study with 44 institutionalized elderly, also detecting a partial recovery of thymulin activity after *in vitro *zinc supplementation. In the same study, a 16 week crossover with 8 weeks of zinc supplementation (20 mg/day) and 8 weeks of placebo caused an increase in serum levels of active thymulin, but the effect was only significant in lean subjects with a body mass index ≤21 [136]. In another *in vivo *study with zinc deficient elderly subjects, zinc supplementation also significantly increased serum thymulin activity [115].

The results showing an improvement of T cell-dependent reactions after zinc supplementation are not unchallenged. In a well designed study, Bogden and coworkers supplemented elderly subjects with zinc in three groups: placebo, 15 mg zinc per day, and 100 mg zinc per day. To prevent underlying effects of deficiencies in other micronutrients, multivitamins and mineral supplements were given to all participants. Baseline data at the beginning of the study [123] as well as results after three months [137] and after one year were reported [138]. After three months, no significant effects were found in response to either dose of zinc, neither on DTH, nor lymphocyte proliferation to several antigens. Initially, zinc supplementation in subjects who are not zinc deficient may be beneficial, but the effect could be only temporary, due to adaptation to a higher zinc intake [137]. This assumption is supported by the observation that NK cell activity increased transiently after 3 months in the group receiving 100 mg zinc, but not after 6 or 12 months. After one year, an increase in DTH was observed in all three groups. This may have been caused by repeated testing, as discussed above, or by a booster effect of the additional multivitamin and mineral supplement that had been administered to all participants. However, zinc supplementation in both groups significantly diminished this effect. The difference between this study and the ones discussed above could be due to the fact that this is the only one that used a placebo group for comparison, or that zinc may interfere with the beneficial effect of one of the other micronutrients, or be a sign of adaptation to zinc supplementation during the longer supplementation period. It has also to be considered that no zinc deficiency was observed in these subjects, which had a mean of approximately 13 μM plasma zinc [138].

A six month, placebo controlled supplementation study with 15 and 30 mg Zn per day (as gluconate) investigated the long-term effects on the immune status of 93 healthy Irish individuals between 55 and 70 years [139]. At baseline, positive correlations between erythrocyte zinc and the amount of T lymphocytes (CD3+), NKT cells (CD3+/CD16+/CD56+), activated T cells (CD25+ HLA-DR+), and naïve T cells (CD3+/CD45RA+) were observed. In addition, erythrocyte zinc was inversely correlated with granulocyte phagocytic capacity and serum zinc with the concentration of CRP [134]. After receiving zinc, the participants supplemented with 15 mg Zn/day had an increased ratio of helper to cyctotoxic T cells, and after 3 months B cell numbers were lower in the 30 mg group compared to the other two groups. Zinc supplementation had no impact on a vast number of other parameters investigated, including inflammation markers, granulocyte phagocytosis, and cytokine production by monocytes. The population investigated in this study had mean serum zinc of 13 μM and thus no zinc deficiency at the beginning of the study, which may explain the lack of significant long-term effects of zinc supplementation on most immune parameters [134,139].

Zinc supplementation does not just promote the immune response; it rather normalizes immune function on the cellular level. Compared to younger subjects, PBMC from elderly have increased *ex vivo *generation of pro-inflammatory cytokines, and normalized cytokine production was observed after zinc supplementation [128,140]. In addition, zinc supplementation improves T cell function, causing reduced levels of unspecifically activated T cells [141], and improved IL-2 mRNA expression and T cell response to stimulation with mitogens [128,140]. This does not indicate, however, that an effect of zinc supplementation on cytokine production is limited to the elderly. Zinc supplementation to younger subjects (19–31 years of age, 15 mg Zn per day as ZnSO_4_), resulted in increased mRNA production of TNF-α and IL-1β in LPS-treated monocytes and granulocytes, and augmented IFN-γ mRNA in T cells treated with microbeads to simulate antigen presentation [142].

The intake of zinc was positively correlated with the results of tests for cognitive performance in 260 subjects between 65 and 90 years [143]. Another study reported a negative correlation between zinc status and indicators for stress and depression and a positive correlation with the mental capacity in elderly from different European countries [144], but this was not confirmed in an investigation of 387 participants between 55 and 87 years who had been supplemented either with placebo, 15, or 30 mg elemental zinc per day (as gluconate) for 6 months [145]. Here, despite significant changes in serum zinc, almost no significant associations between zinc status at baseline and eight measures of cognitive performance were found. In response to supplementation, only two statistically significant effects were observed, namely a temporary improvement of spatial working memory and an impairment of attention [145].

On the other hand, another recent study with 97 healthy elderly from Italy, Greece, and Poland found slight beneficial effects of zinc supplementation on cognitive performance, measured by the Mini Mental State examination, and mood conditions, measured by the geriatric depression scale. Furthermore, it demonstrated an improvement on the perceived stress scale. Notably, this latter effect of zinc supplementation was more pronounced in subjects with a certain polymorphism in the promoter region of the gene for IL-6 [146].

Inflammatory cytokines have been suggested to affect cognitive performance via the production of reactive oxygen species in brain ageing [147]. Chronic low level inflammation is common in the elderly, and zinc deficiency impairs cytokine homeostasis in this population, leading to increased production of pro-inflammatory cytokines such as IL-6, which can be corrected by zinc supplementation [70,140]. Taken together, these data suggest that a supplementation with zinc could act on cognitive and psychological parameters via modulation pro-inflammatory cytokine levels, although more data to confirm this hypothesis are certainly required.

Other studies investigated zinc supplementation in combination with additional micronutrients. In a larger study, 725 institutionalized patients (65–103 years) were supplemented for 2 years with zinc sulfate (20 mg zinc) together with selenium sulfide (100 μg selenium), or multivitamins, or a combination of both [148]. Patients treated with selenium and zinc either alone or together with vitamins, showed higher antibody titers after influenza vaccination, whereas vitamins alone had a negative effect on response to vaccination. An improvement of influenza vaccination response by zinc was not confirmed in another study in which zinc was administered together with arginine [149], but the relatively high dose of zinc used in this case (zinc sulfate, 400 mg per day = 90 mg elemental zinc) might have suppressed T cell help.

Although supplementation together with other micronutrients makes it difficult to specify the contribution of zinc, this is possible if appropriate controls are included. The example of a recent study in Mexican children clearly demonstrates an effect of zinc supplementation on several parameters of immune function even if it was administered in the presence of other micronutrients [150].

Zinc is generally regarded as a non-toxic essential metal. Accordingly, correction of zinc deficiency in the elderly should generally improve the performance of the immune system, but overdosing zinc supplementation can also have a negative impact on immune efficiency. In this respect, two effects are relevant. On the one hand, zinc can interfere with the uptake of copper. Hence, long-term high-dose zinc supplementation can lead to severe anemia and neutropenia, based on copper deficiency [151]. On the other hand, pharmacological doses of zinc suppress T cell-dependent immune responses [51], and may cause a temporary reduction of B cell counts [134], leading to an impaired adaptive immune response when too much zinc is supplemented.

## Conclusion

Zinc ions are indispensable for immune function, especially for T cell mediated events, which are primarily affected in immunosenescence. The high prevalence of zinc deficiency in hospitalized subjects and the correlation between zinc status and immune function surely justifies zinc supplementation to these patients to normalize zinc levels, and hereby restore important functions of the immune system. One central question remains: Should the decrease of zinc status with age be seen as a marginal zinc deficiency, which, in combination with multiple other factors, increases the susceptibility for infectious diseases and cancer, and should zinc be given to those with no clinical symptoms? From the results published so far, it looks like a moderate zinc supplementation that stays well below the limits for adverse effects could have substantial benefits. However, a rapid and reliable method for the assessment of zinc status would be helpful to identify those who would benefit most from zinc supplementation.

## Competing interests

The authors declare that they have no competing interests.

## Authors' contributions

HH and LR have written the manuscript.
